# Survival trends over 20 years in patients with advanced cholangiocarcinoma: Results from a national retrospective analysis of 922 cases in Italy

**DOI:** 10.3389/fonc.2023.1128930

**Published:** 2023-04-05

**Authors:** Andrea Casadei-Gardini, Francesco Leone, Giovanni Brandi, Mario Scartozzi, Nicola Silvestris, Daniele Santini, Luca Faloppi, Massimo Aglietta, Maria Antonietta Satolli, Alessandro Rizzo, Sara Lonardi, Giuseppe Aprile, Lorenzo Fornaro

**Affiliations:** ^1^ Department of Oncology, Vita-Salute San Raffaele University, IRCCS San Raffaele Scientific Institute Hospital, Milan, Italy; ^2^ Division of Medical Oncology, ASL BI, Nuovo Ospedale degli Infermi, Ponderano, BI, Italy; ^3^ Oncology Unit, Department of Experimental, Diagnostic and Specialty Medicine, Sant’Orsola-Malpighi Hospital, Bologna, Italy; ^4^ Medical Oncology, University Hospital and University of Cagliari, Cagliari, Italy; ^5^ Medical Oncology Unit, Department of Human Pathology “G. Barresi”, University of Messina, Messina, Italy; ^6^ UOC Oncologia Medica, “Sapienza University”, Polo Pontino, Rome, Italy; ^7^ Macerata General Hospital, Medical Oncology Unit, Macerata, Italy; ^8^ Division of Medical Oncology, Candiolo Cancer Institute, FPO - IRCCS, Candiolo, Italy; ^9^ Division of Medical Oncology 1, Centro Oncologico Ematologico Subalpino, Azienda Ospedaliero-Universitaria Città della Salute e della Scienza di Torino, Torino, Italy; ^10^ Struttura Semplice Dipartimentale di Oncologia Medica per la Presa in Carico Globale del Paziente Oncologico “Don Tonino Bello”, I.R.C.C.S. Istituto Tumori “Giovanni Paolo II”, Bari, Italy; ^11^ Oncology Unit 3, Department of Oncology, Veneto Institute of Oncology IOV - IRCCS, Padua, Italy; ^12^ Department of Oncology, San Bortolo General Hospital, Vicenza, Italy; ^13^ U.O. Medical Oncology 2 University, Azienda Ospedaliero-Universitaria Pisana, Pisa, Italy

**Keywords:** advanced cholangiocarcinoma, retrospective study, Italy, outcomes, survival

## Abstract

Cholangiocarcinoma is a rare group of tumors that involve the hepatic biliary tree. Prognosis for patients with cholangiocarcinoma remains dismal. Herein, we present survival trends over a long time period spanning almost 20 years in patients with advanced cholangiocarcinoma receiving systemic chemotherapy. We retrospectively analyzed a large multicenter dataset of cholangiocarcinoma outpatients evaluated in 14 centers within the Cholangiocarcinoma Italian Group Onlus (Gruppo Italiano Colangiocarcinoma Onlus, G.I.C.O.) between 2000 and 2017 (first-line), and 2002 and 2017 (second-line). Three time periods were considered: 2000-2009, 2010-2013, and 2014-2017. A total of 922 patients (51.19% male) with cholangiocarcinoma undergoing first-line therapy were evaluated. The median durations of follow-up for progression-free survival (PFS) and overall survival (OS) were 37 and 57 months, respectively. PFS at 12 months in the three periods of starting first-line therapy was similar, ranging from 11.71% to 15.25%. OS at 12 months progressively improved (38.30%, 44.61% and 49.52%, respectively), although the differences were not statistically significant after adjusting for age, disease status, and primary tumor site. A total of 410 patients (48.5% male) underwent second-line chemotherapy. The median durations of follow-up for PFS and OS were 47.6 and 41.90 months, respectively. An OS of 24.3%, 32.3%, and 33.1% was observed in 2002-2009, 2010-2013, and 2014-2017, respectively. Despite incremental benefits across years, our clinical experience confirms that modest overall advances have been achieved with first- and second-line chemotherapy in advanced cholangiocarcinoma. Efforts should focus on the identification of patients who derive the greatest benefit from treatment.

## 1 Introduction

Cholangiocarcinoma is a rare group of tumors that involve the hepatic biliary tree (1). Cholangiocarcinomas account for about 2% of all gastrointestinal malignancies and are the second most common hepatic tumor following hepatocellular carcinoma (2). The incidence of these tumors is thought to be increasing in recent decades, at least in Western countries, which is mostly related to an increase in intrahepatic cholangiocarcinoma (3, 4). Cholangiocarcinomas are classified according to their anatomical location as intrahepatic and extrahepatic, with tumors in the latter category further divided into perihilar or distal (1, 3). About half of cholangiocarcinomas are perihilar, while smaller proportions have distal and intrahepatic locations (5). Risk factors for cholangiocarcinoma include primary sclerosing cholangitis, choledochal cysts, parasitic infections, inflammatory bowel disease, diabetes, obesity, and alcohol use (1, 3). The prognosis for patients with cholangiocarcinoma is dismal, with a 5-year overall survival (OS) of <20% (6). This is also related to the fact that these tumors are usually diagnosed at advanced stages and surgery with curative intent and adjuvant therapy is only feasible for a small proportion of patients.

Treatment is driven by surgical management whenever possible depending on the site of the tumor, although few patients are candidates for a hepatic graft (1). If a patient is ineligible for surgery or in case of recurrence, chemotherapy can be considered (1). Based on the results of the ABC-02 trial, combination of cisplatin plus gemcitabine (CG) was established as first-line therapy for patients with advanced cholangiocarcinoma (7). Patients with disease progression following cisplatin plus gemcitabine typically have poor OS of less than 6 months (8). Recently, the results of the TOPAZ-1 trial investigating gemcitabine/cisplatin plus durvalumab appear to be promising (9, 10). This phase 3 randomized, double-blind, placebo-controlled study randomized patients to CG plus durvalumab for eight cycles followed by durvalumab only or placebo. Median OS was 12.8 months vs. 11.5 months (hazard ratio [HR], 0.80; 95% confidence interval [CI], 0.66–0.97; p = 0.021), median PFS was 7.2 months vs. 5.7 months (HR, 0.75; 95% CI, 0.64–0.89; p = 0.001), and ORR (26.7% vs. 18.7%) was superior with gemcitabine/cisplatin plus durvalumab compared to gemcitabine/cisplatin plus placebo. These results are encouraging and will lead to a change in first-line standard, paving the way toward other combinations and translational research in the near future.

The role of second-line chemotherapy after progression on cisplatin and gemcitabine remains debatable, with very limited clinical data (11). Recently, however, the ABC-06 trial reported that as second-line chemotherapy the FOLFOX (folinic acid, fluorouracil, and oxaliplatin) regimen is associated with improved OS in patients with advanced cholangiocarcinoma (12). In particular, at 12 months, the rate of OS was in patients receiving active symptom control was 11.4% compared to 25.9% in those receiving active symptom control and FOLFOX. Accordingly, it was proposed that FOLFOX should be the standard for second-line chemotherapy in patients with advanced cholangiocarcinoma. This conclusion has been reinforced after the recent presentation of quality of life (QoL) result for ABC-06, showing that active chemotherapy does not impact negatively on QoL measures (13). Moreover, alternative combinations with fluorouracil and liposomal irinotecan (Nal-Iri) did not confirm preliminary findings of potential benefit among pretreated patients (14–16), confirming that further studies are warranted in order to optimize systemic treatment beyond first-line progression.

Today, molecular biology and next-generation sequencing techniques are playing an increasingly important role in choice of therapy. In fact, the ESMO Precision Medicine Working Group has recommended that clinical research centers adopt multigene sequencing to screen patients who are eligible for clinical trials and to accelerate drug development, as well as prospectively collect data that may provide additional information on the use of this methodology in the future (17). For cholangiocarcinomas in particular, molecular techniques can evaluate the possibility to target actionable genomic alterations with approved agents such as include ivosidenib for tumors harboring IDH1 mutations, and infigratinib and pemigatinib for those with FGFR2 fusions (18). Ivosidenib, for example, has been associated with a favorable benefit for OS vs placebo in patients whose tumor harbors aberrations in IDH1 (19). Similarly, pemigatinib appears to be beneficial in previously treated patients with cholangiocarcinoma with FGFR2 fusions or rearrangements (20).

Analysis of survival trends over recent decades is important in order to understand the efficacy of chemotherapy in patients with advanced cholangiocarcinoma. Herein, we present survival trends over a long time period spanning almost 20 years in patients with advanced cholangiocarcinoma receiving systemic chemotherapy. The analysis was also divided into different time frames to better understand differences in survival following the introduction of cisplatin plus gemcitabine as a preferred regimen for first-line chemotherapy.

## 2 Materials and methods

### 2.1 Patient selection

We retrospectively analyzed a large multicenter dataset of cholangiocarcinoma outpatients evaluated in 14 centers within the Cholangiocarcinoma Italian Group Onlus (Gruppo Italiano Colangiocarcinoma Onlus, G.I.C.O.) between 2000 and 2017 (first-line), and 2002 and 2017 (second-line). Selection criteria included histological or cytological diagnosis of biliary tract cancer, treatment with systemic chemotherapy for advanced disease, availability of clinical data and follow up information and written informed consent for data collection.

### 2.2 Endpoints and outcomes

Two primary outcomes were analyzed, namely PFS and OS. PFS and OS time were calculated from the start date of systemic therapy to the date of progression or death, respectively. Event-free time was calculated up to the date of last clinical visit. Age was categorized using 60 and 70 years as cut-offs, while clinical sensitivity variables were defined as follows: sensitive (progression after 90 days of day 1 of the last cycle of first­line cisplatin and gemcitabine), or resistant (progression within the first 90 days after completion of day 1 of the last cycle of first­line cisplatin and gemcitabine). Second-line therapies were grouped as COMBINED (patients had received one of cisplatin + gemcitabine, oxaliplatin + gemcitabine, gemcitabine plus capecitabine or other combinations), FOLFOX - FOLFIRI or MONO (patients had received gemcitabine or fluoropyrimidine monotherapy).

### 2.3 Statistical analysis

Continuous variables are presented as means with standard deviations (SD) and categorical variables as number of subjects and percentage. The Kaplan-Meier method was used to estimate PFS and OS at 12 and 18 months. To evaluate the effect of demographic and clinical characteristics on PFS and OS, univariate analysis was performed by the Cox Proportional-Hazards model. The time at which patients reached progression or death was compared according to year of starting the first- or second-line therapy (grouped by ranges: 2000-2009, 2010-2013, and 2014-2017).

Moreover, an interaction test was used to assay whether the PFS and OS were different in the three periods at which patients received first- or second-line therapy according to the age and therapy type. A subgroup analysis was performed for significant interactions. All Cox Proportional-Hazards models were adjusted for disease status, primary tumor site, and time in first-line therapy (when appropriate). The hazard ratios (HR) associated with the PFS and OS were calculated with their 95% confidence interval (CI) for each factor from the Cox Proportional-Hazards model. The Likelihood Ratio test was used as a test of statistical significance and p-values were adjusted for multiple comparisons by using the Holm correction method. Differences, with a p-value less than 0.05, were considered as significant. Data were analyzed using R v4.1.1 software.

## 3 Results

### 3.1 First-line therapy

A total of 922 patients (472 males, 51.19%) with cholangiocarcinoma were evaluated. Of these, 796 died (86.33%) and 843 experienced disease progression (91.43%). Demographic and clinical characteristics of the study participants are summarized in **Table 1**. The mean age at first-line therapy was 64.79 years. Half of patients (50.0%) had an intrahepatic primary tumor, while 22.1%, 21.4%, and 6.1% had extrahepatic, gallbladder, and ampullary primary tumors, respectively. Details on the first-line therapy received in the different time periods are reported in **Table 2**, which shows a progressive increase in use of cisplatin + gemcitabine. The median durations of follow-up for PFS and OS were 37 and 57 months, respectively. PFS was 13.90% and 7.07% at 12 and 18 months, respectively; OS was 43.90% and 26.00% at 12 and 18 months, respectively.

**Table 1:** Demographic and clinical characteristics of study participants at baseline.

Characteristic	N (%) N=922
Gender	
Male	472 (51.19%)
Female	450 (48.81%)
Age at first-line therapy years	64.79 (9.99)
Not reported	3 (0.33%)
Primary tumor Site	
Intrahepatic	461 (50%)
Extrahepatic	204 (22.13%)
Gallbladder	197 (21.37%)
Ampullary tumor	56 (6.07%)
Not reported	4 (0.43%)
Disease Status	
Locally Advanced	207 (22.45%)
Metastatic	709 (76.9%)
Not reported	6 (0.65%)
Period of first-line therapy	
2000-2009	274 (29.72%)
2010-2013	374 (40.56%)
2014-2017	272 (29.5%)
Not reported	2 (0.22%)

**Table 2:** Types of first-line chemotherapy administered.

Type	Period		
	2000-2009	2010-2013	2014-2017
Cisplatin + gemcitabine	0 (0%)	98 (26.20%)	94 (34.56%)
GEMOX	128 (46.72%)	139 (37.17%)	85 (31.25%)
Gemcitabine	88 (32.12%)	80 (21.39%)	59 (21.69%)
FOLFOX/CAPOX	13 (4.75%)	20 (5.35%)	9 (3.31%)
Fluoropyrimidine monotherapy	5 (1.83%)	11 (2.94%)	12 (4.41%)
Other	40 (14.60%)	26 (6.95%)	13 (4.78%)

GEMOX, gemcitabine + oxaliplatin; FOLFOX, 5-fluorouracil/leucovorin + oxaliplatin; CAPOX, capecitabine + oxaliplatin.

#### 3.1.1 Progression-free survival

**Figure 1:**
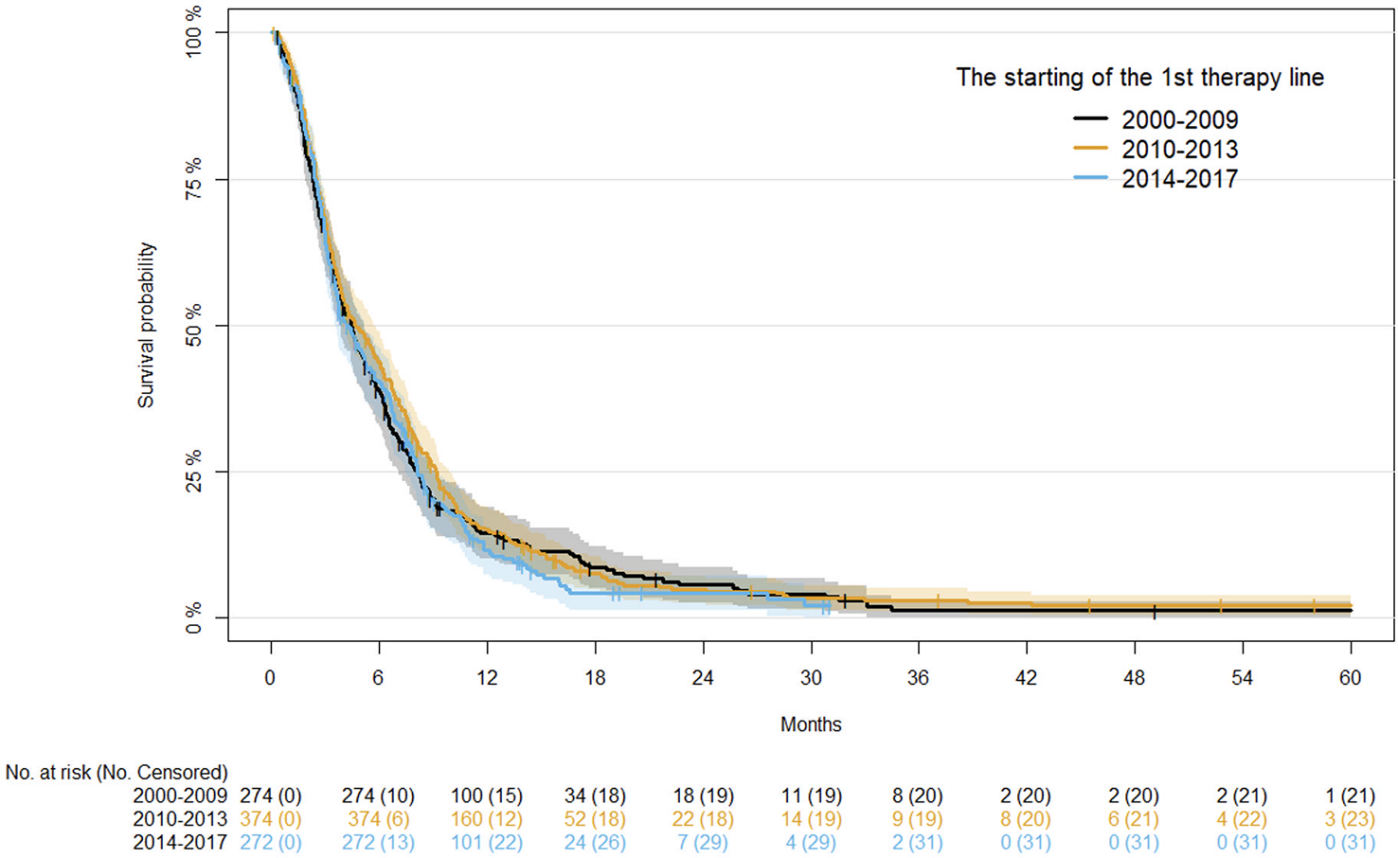
Kaplan-Meier curves for PFS after first-line therapy.

Univariate analysis of demographics and clinical factors on PFS is reported in **Table S1**. In detail, PFS at 12 months in the three periods of starting first-line therapy was similar, ranging from 11.71% to 15.25%. No significant association between time range of starting first-line therapy and PFS was observed after adjusting for age, disease status, and primary tumor site (**Table 3**). Kaplan-Meier curves for PFS by period of first-line therapy are shown in **Figure 1**. 

**Table 3:** Cox model after adjusting for confounding factors on PFS for first-line therapy (N=922).

Characteristic	HR (95% CI)	p-value
Start of first-line therapy		0.7059
2000-2009	1	
2010-2013	0.92 (0.78: 1.09)	
2014-2017	1.03 (0.86: 1.23)	
Age at first-line therapy	1 (0.99: 1.01)	0.7059
Primary tumor site		0.0092
Intrahepatic	1	
Extrahepatic	0.88 (0.74: 1.05)	
Gallbladder	1.18 (0.99: 1.41)	
Ampullary tumor	0.69 (0.51: 0.94)	
Disease status		0.0835
Locally-advanced	1	
Metastatic	1.2 (1.02: 1.42)	

#### 3.1.2 Overall survival

**Figure 2:**
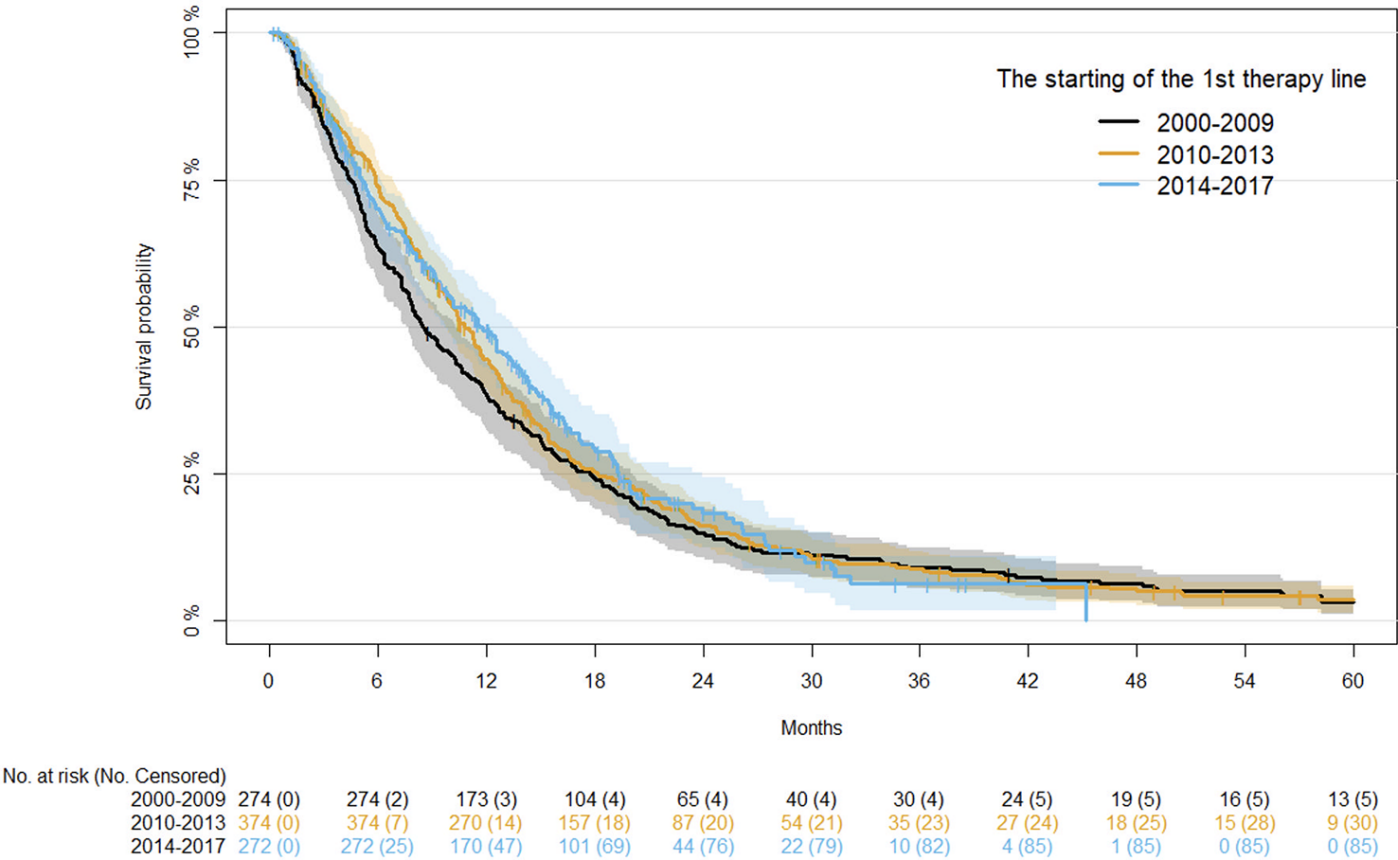
Kaplan-Meier curves for OS after first-line therapy.

For OS, univariate analysis of demographics and clinical factors is reported in **Table S2**. Considering the first period (2000-2009), moving to the more recent period (2014-2017), OS at 12 months progressively improved (38.30%, 44.61% and 49.52%, respectively), although the differences were not statistically significant after adjusting for age, disease status, and primary tumor site (**Table 4**). Kaplan-Meier survival curves for OS in the different time periods are shown in **Figure 2**.

**Table 4:** Cox model after adjusting for confounding factors on OS for first-line therapy (N=922).

Characteristic	HR (95% CI)	p-value
Start of first-line therapy		0.1851
2000-2009	1	
2010-2013	0.89 (0.76: 1.05)	
2014-2017	0.84 (0.7: 1.02)	
Age at first-line therapy	1.01 (1: 1.02)	0.0187
Primary tumor site		0.0005
Intrahepatic	1	
Extrahepatic	1.05 (0.87: 1.26)	
Gallbladder	1.44 (1.20: 1.72)	
Ampullary tumor	0.77 (0.57: 1.05)	
Disease status		0.0051
Locally-advanced	1	
Metastatic	1.31 (1.10: 1.57)	

### 3.2 Second-line therapy

A total of 410 patients (199 male,48.5%) were evaluated in this analysis. The median durations of follow-up for PFS and OS were 47.6 and 41.90 months, respectively. PFS was 5.94% and 2.83% at 12 and 18 months, respectively; OS was 29.60% and 17.80% at 12 and 18 months, respectively. Demographic and clinical characteristics of study participants are summarized in **Table 5**. Mean age at second-line therapy was 64.19 years. The majority of patients (53.6%) had an intrahepatic primary tumor, while 20.6%, 18.9%, and 6.6% had an extrahepatic, gallbladder and ampullary primary tumor, respectively.

**Table 5:** Demographic and clinical characteristics of study participants undergoing second-line therapy at baseline (n= 410).

Characteristic	Overall
Death	
No	52 (12.78%)
Yes	355 (87.22%)
Progression of disease	
No	19 (4.63%)
Yes	369 (90%)
Not reported	22 (5.37%)
Gender	
Male	199 (48.54%)
Female	211 (51.46%)
Age at second-line therapy (years)	64.19 (9.75)
Age range at second-line therapy (years)	
≤60	135 (33.17%)
61 to 70	145 (35.63%)
≥71	127 (31.2%)
Clinical sensitivity	
No	133 (32.44%)
Resistant (PD within 90 days)	55 (13.41%)
Sensitive (PD after 90 days)	221 (53.9%)
Not reported	1 (0.24%)
Primary tumor site	
Intrahepatic	218 (53.56%)
Extrahepatic	84 (20.64%)
Gallbladder	77 (18.92%)
Ampullary tumor	27 (6.63%)
Not reported	1 (0.25%)
Biliary drainage	
No	328 (80.59%)
Yes	79 (19.41%)
Surgery on primary tumor	
No	209 (51.35%)
Yes	198 (48.65%)
ECOG PS	
0	180 (44.23%)
1	190 (46.68%)
2	10 (2.46%)
3	2 (0.49%)
Not reported	25 (6.14%)
Disease status	
Locally advanced	87 (21.38%)
Metastatic	319 (78.38%)
Not reported	1 (0.25%)
Type of first-line therapy	
Cisplatin + gemcitabine	99 (24.32%)
GEMOX	177 (43.49%)
Gemcitabine	64 (15.72%)
FOLFOX/CAPOX	21 (5.16%)
Fluoropyrimidine monotherapy	8 (1.97%)
Other	38 (9.34%)
Time in first-line therapy (months)	8.21 (6.08)
Not reported	1 (0.24%)
Period of second-line therapy	
2002-2009	112 (27.52%)
2010-2013	145 (35.63%)
2014-2017	150 (36.86%)
Type of second-line therapy	
FOLFOX/CAPOX	24 (5.9%)
FOLFIRI	65 (15.97%)
Cisplatin + gemcitabine	25 (6.14%)
GEMOX	28 (6.88%)
Gemcitabine	47 (11.55%)
Fluoropyrimidine monotherapy	117 (28.75%)
Gemcitabine + capecitabine	41 (10.07%)
Other	60 (14.74%)
Group of second-line therapy	
COMBINED	154 (37.84%)
FOLFOX or FOLFIRI	89 (21.87%)
MONO	164 (40.29%)
Response	
CR	11 (2.7%)
PR	84 (20.64%)
SD	128 (31.45%)
PD	184 (45.21%)

COMBINED, includes the following combination regimens: Cisplatin + gemcitabine, GEMOX, Gemcitabine + capecitabine and other; FOLFOX, 5-fluorouracil/leucovorin + oxaliplatin; FOLFIRI, 5-fluorouracil/leucovorin + irinotecan; MONO, includes the following agents administered as monotherapy: gemcitabine or fluoropyrimidine.

Results are expressed as mean with standard deviation or as number of subjects with percentage.

#### 3.2.1 Progression-free survival

**Figure 3:**
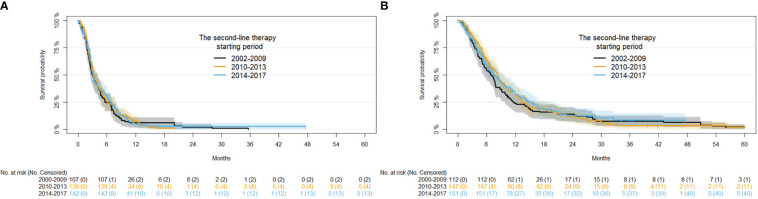
Kaplan-Meier curves for PFS **(A)** and OS **(B)** for second-line therapy by time period.

Univariate of demographic and clinical factors on PFS are reported in **Table S3**. Regarding the period of second-line therapy, a PFS of 6.43%, 7.92% and 5.30% at 12 months was observed in 2002-2009, 2010-2013, and 2014-2017, respectively. Of note, PFS at 12 months was modest in the FOLFOX or FOLFIRI groups compared with that in the COMBINED and MONO therapy groups (4% and 7%, respectively). No significant association among covariates and PFS was observed after adjusting for disease status and period of first-line therapy (**Table S3**). Kaplan-Meier curves for PFS in the different time periods of second-line therapy are presented in **Figure 3A**. No significant interaction between the time period of second-line therapy and type of second-line therapy (p-value for interaction = 0.2840) was observed. While there was significant interaction between the second-line therapy starting period and the age at second-line therapy (p-value for interaction = 0.0286).

**Figure 4:**
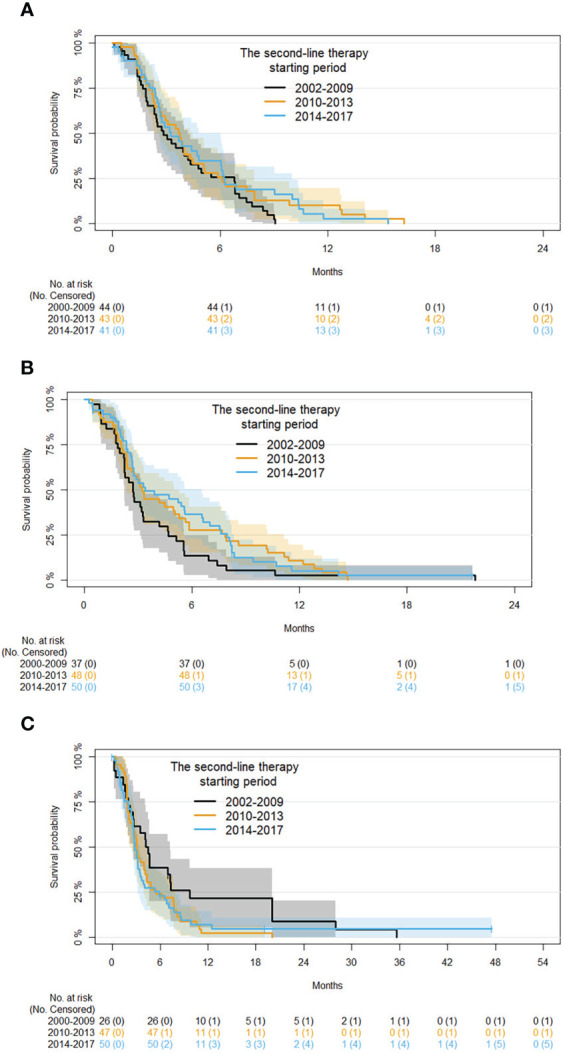
Kaplan-Meier curves for PFS by period of second-line therapy and age (subgroup analysis on patients with age ≤60, 61 to 70, and ≥71 years are shown in panels **(A-C)**, respectively).

Subgroup analysis by age at second-line therapy and adjusting for disease status and time in first-line therapy are reported in **Table 6**. For patients with an age ≤60 years, compared with those starting therapy in 2002-2009 a 28% and 19% risk reduction of progression, respectively, was found in those starting second-line therapy in 2010-2013 (HR 0.72, 95% CI 0.45: 1.14) and 2014-2017 (HR 0.81, 95% CI 0.5: 1.32). For patients with an age between 61 and 70 years, a 56% and 30% risk reduction of progression, respectively, was also estimated in patients starting second-line therapy in 2010-2013 (HR 0.54, 95% CI 0.54 0.34: 0.86) and 2014-2017 (HR 0.7, 95% CI 0.44: 1.11). In patients with an age ≥71 years, the trend was inverted. In particular, comparing with those starting therapy in 2002-2009 a 39% and 47% risk improvement of progression was found in patients starting second-line therapy in 2010-2013 (HR 1.39, 95% CI 0.82: 2.37) and 2014-2017 (HR 1.47, 95% CI 0.86: 2.53). Kaplan-Meier curves for PFS with patients grouped by age in the different time periods are shown in **Figure 4**.

**Table 6:** Subgroup analysis of the period of second-line therapy on PFS by age.

Characteristic	Age at second-line therapy								
	≤60 years (n=128)			61 to 70 years (n=135)			≥71 years (n=123)		
	Overall Events (N)	HR (95%CI)	p-value	Overall Events (N)	HR (95%CI)	p-value	Overall Events (N)	HR (95%CI)	p-value
**Period of second-line therapy**			0.3705			0.0355			0.3384
2002-2009	43(44)	1		37(37)	1		25(26)	1	
2010-2013	41(43)	0.72 (0.45: 1.14)		47(48)	0.54 (0.34: 0.86)		46(47)	1.39 (0.82: 2.37)	
2014-2017	38(41)	0.81 (0.5: 1.32)		45(50)	0.7 (0.44: 1.11)		45(50)	1.47 (0.86: 2.53)	
**Second-line therapy**			0.2375			0.6923			0.5099
COMBINED	47(49)	1		54(54)	1		40(42)	1	
FOLFOX or FOLFIRI	38(39)	1.14 (0.73: 1.79)		31(32)	0.92 (0.58: 1.44)		16(16)	1.34 (0.73: 2.45)	
MONO	37(40)	1.47 (0.94: 2.31)		44(49)	0.9 (0.6: 1.35)		60(65)	1.07 (0.7: 1.62)	
Disease Status			0.6658			0.1386			0.2677
Locally Advanced	30(30)	1		22(24)	1		30(31)	1	
Metastatic	92(98)	0.90 (0.58: 1.4)		107(111)	1.13 (0.69: 1.85)		86(92)	1.22 (0.77: 1.95)	
**Time in first-line therapy (months)**	122(128)	1.00 (0.96: 1.03)	0.7188	129(135)	0.92 (0.89: 0.95)	<0.0001	116(123)	0.95 (0.92: 0.98)	0.0005

COMBINED, includes the following combination regimens: Cisplatin + gemcitabine, GEMOX, Gemcitabine + capecitabine and Others; FOLFOX, 5-fluorouracil/leucovorin + oxaliplatin; FOLFIRI, 5-fluorouracil/leucovorin + irinotecan; MONO, includes the following agents administered as monotherapy: Gemcitabine or Fluoropyrimidine. p-value: Likelihood Ratio test p-value.

#### 3.2.2 Overall survival

Univariate analysis of demographic and clinical factors on OS are reported in **Table S4**. Regard the time period of second-line therapy, an OS of 24.3%, 32.3%, and 33.1% was observed in 2002-2009, 2010-2013, and 2014-2017, respectively. OS at 12 months was lower in FOLFOX or FOLFIRI (26.7%) and MONO (25.0%) groups compared with the COMBINED therapy groups (37.4%). No significant association among covariates and OS were observed after adjusting for disease status and period of second-line therapy (**Table S4**). Kaplan-Meier survival curves for the OS in the starting of the second-line therapy periods are shown in **Figure 3B**. An interaction between period of second-line therapy and OS was not observed for the different age groups or type of second-line therapy (p-values for interaction: 0.0713 and 0.1597, respectively).

## 4 Discussion

The present analysis of patients with advanced cholangiocarcinoma undergoing first- and second-line chemotherapy compared outcomes during three time periods, 2000-2009, 2010-2013, and 2014-2017. As expected, we observed a shift in the use of cisplatin plus gemcitabine after 2010, following the publication of the ABC-02 trial (7), which was administered to 26% of patients in 2010-2013 and to 35% in 2014-2017. PFS did not show a significant association with the time period of starting first-line therapy, but the high percentage of patients receiving a treatment other than cisplatin + gemcitabine and the retrospective nature of the analyses could partly explain this finding. However, for OS at 12 months a trend was observed towards increased survival at later time periods, increasing progressively from 38.3% in 2000-2009 to 49.5% in 2014-2017. Despite a more than 10% increase in OS, the differences did not reach statistical significance after adjusting for confounding factors. However, it should be pointed out that only about one-third of patients received cisplatin plus gemcitabine, and broadly similar proportions received GemOx or gemcitabine alone. Taken together, our results confirm that CG rapidly entered the clinical scenario after publication of ABC-02, even though a large number of patients still receive regimens different from CG (possibly due to suboptimal general conditions, physician preference or other reasons not captured by our database). Moreover, we confirm that alternative options beyond CG are eagerly needed in advanced cholangiocarcinoma: in this regard, we are conducting an observational study within the G.I.C.O. group with the aim of estimating the real-world impact of the addition of durvalumab to first-line CG compared to the TOPAZ-1 study.

For the group of patients undergoing second-line chemotherapy, in line with the limited clinical evidence available at the time of treatment initiation, a wide variety of regimens were administered. The most widely administered regimen was fluoropyrimidine monotherapy, although FOLFOX or FOLFIRI had been administered to about one-fifth of patients. Response rates were low and 45.2% of the patients showed progressive disease following second-line chemotherapy. Overall, our data indicate the limited value of second-line chemotherapy in a large proportion of patients, as previously highlighted (21).

In our analysis, PFS and OS were numerically higher in patients receiving one of the COMBINED (cisplatin + gemcitabine, gemox, gemcitabine + capecitabine or others) therapies compared to FOLFOX or FOLFIRI or monotherapy, although the differences did not reach statistical significance. Of note, OS at 12 months in ABC-02 was 25.9% (7) compared to 33.1% in the last time period analyzed in the present study. Thus, we conclude that, even if the benefits of second-line therapy are limited among unselected patients in the real-world practice, they might be significant in some subgroups of patients (22, 23). Interestingly, subgroup analysis by age at second-line therapy showed that in patients ≤60 and 61-70 years, compared with those starting therapy in 2002-2009 risk reduction of progression was found in those starting second-line therapy in 2010-2013 and 2014-2017, while the opposite was seen in patients ≥71 years. Therefore, even if a definitive conclusion about the optimal management of pre-treated elderly patients with cholangiocarcinoma cannot be derived from our analysis, overall benefit of second-line therapies should not be overestimated and some caution is warranted when facing frail patients with pre-treated disease (for whom active symptoms control is still an option on the basis of ABC-06). Median age was 65 years but upper age limit was as high as 84 years in ABC-06: since subgroup analysis according to age was not presented in the publication, it could be of interest to better explore the actual impact on OS and QoL of salvage chemotherapy for these higher-risk patients.

Similar to first-line chemotherapy, a difference in OS at 12 months was apparent in the three time periods, increasing from 24.3% in 2002-2009 to 33.1% in 2014-2017, although these differences did not reach statistical significance. Taken together, our data confirm the findings of randomized trials, such ABC-02 and others (14–16). This is relevant since real-world outcomes appear to be similar to those seen in clinical trials (24), although prognosis still remains dismal (25).

Cholangiocarcinoma is emerging as a promising candidate for precision medicine in light of the multiple potentially targetable molecular alterations identified, especially for the intrahepatic subset. Among these, FGFR2 rearrangements and IDH1 mutations have already entered the clinical scenario. In our study we did not collect data about molecular profiling, but we can speculate that only a limited proportion of patients treated in more recent years had their disease profiled for druggable alterations and received targeted treatment. Therefore, together with information about the use of durvalumab plus CG in first-line, we are currently collecting data about the prevalence of patients with cholangiocarcinoma assessed for molecular alterations in Italian practice and about the potential impact of target treatment in molecularly-defined patient subsets. Preliminary findings on ivosidenib are encouraging (26).

In summary, in our clinical experience the benefits of first- and second-line chemotherapy appear to be limited in patients with advanced choangiocarcinoma overall, although some impact may be seen in some subgroups of patients that remain to be better defined. Over the years, therapeutic techniques have undoubtedly improved (e.g. for biliary stenting), but at present it is difficult to quantify their impact on outcomes in the absence of specific studies. In line with other reports (27), our study further underlines the aggressiveness of biliary tract cancers, and the need for better treatment options as well as specialized management in trained centers: this could contribute to offering effective palliative measures, with the aim of increasing the chances of receiving active therapeutic options in the advanced setting. Future therapeutic combinations with novel agents and the routine introduction of individualized therapy with targeted agents has the potential to improve overall outcomes in selected subgroups of patients. In this regard, the results of the TOPAZ-1 trial on gemcitabine/cisplatin plus durvalumab are likely to change clinical practice in the near future (9, 10).

## Data availability statement

The original contributions presented in the study are included in the article/**Supplementary Material**. Further inquiries can be directed to the corresponding author.

## Ethics statement

Ethical review and approval was not required for the study on human participants in accordance with the local legislation and institutional requirements. The patients/participants provided their written informed consent to participate in this study.

## Author contributions

LFo and AC-G: creators. All other authors: investigators. All authors contributed to the article and approved the submitted version.
