# Supplementary material for: Survival trends over 20 years in patients with advanced cholangiocarcinoma: Results from a national retrospective analysis of 922 cases in Italy
Source: Front Oncol. 2023 Apr 5;13:1128930. doi: 10.3389/fonc.2023.1128930 (PMC10113474; doi:10.3389/fonc.2023.1128930)
Supplement: Supplementary file 1 [file Table_1.docx]

Supplementary Material

**Supplementary material**

**Table S1**. Contingency tables and summary output of univariate PFS analysis (N=922). p-value: Likelihood Ratio test p-value.

| **Characteristic** | **Overall**  **Events (N)** | **Events (N)**  **at 12**  **months** | **Survival (%)**  **at 12**  **months** | **Events (N)**  **at 18**  **months** | **Survival (%)**  **at 18**  **months** | **HR (95%CI)** | **p-value** |
| --- | --- | --- | --- | --- | --- | --- | --- |
| **Gender** |  |  |  |  |  |  | 0.9999 |
| Male | 431(472) | 388(59) | 14.75% | 414(27) | 7.86% | 1 |  |
| Female | 411(448) | 373(51) | 13.30% | 397(20) | 6.51% | 0.94  (0.82 : 1.07) |  |
| **Age at first-line therapy** | 841(919) |  |  |  |  | 1  (0.99 : 1.01) | 0.9999 |
| **Primary tumor site** |  |  |  |  |  |  | 0.0080 |
| Intrahepatic | 424(460) | 383(55) | 13.95% | 412(20) | 6.22% | 1 |  |
| Extrahepatic | 185(204) | 157(36) | 20% | 172(16) | 10.95% | 0.86  (0.73 : 1.03) |  |
| Gallbladder | 185(196) | 177(11) | 7.08% | 180(6) | 4.90% | 1.2  (1.01 : 1.42) |  |
| Ampullary tumor | 45(56) | 42(7) | 17.49% | 44(5) | 12.83% | 0.71  (0.52 : 0.97) |  |
| **Disease status** |  |  |  |  |  |  | 0.0835 |
| Locally advanced | 188(206) | 165(33) | 18.12% | 178(16) | 10.68% | 1 |  |
| Metastatic | 649(708) | 592(76) | 12.71% | 628(31) | 6.18% | 1.21  (1.03 : 1.42) |  |
| **Period of first-line therapy** |  |  |  |  |  |  | 0.9999 |
| 2000-2009 | 252(274) | 225(34) | 14.66% | 238(18) | 8.83% | 1 |  |
| 2010-2013 | 349(374) | 310(52) | 15.25% | 334(22) | 7.84% | 0.93  (0.79 : 1.1) |  |
| 2014-2017 | 241(272) | 226(24) | 11.71% | 239(7) | 4.53% | 1.04  (0.87 : 1.25) |  |

**Table S2**. Contingency tables and summary output of univariate OS analysis (N=922). p-value: Likelihood Ratio test p-value.

| **Characteristic** | **Overall**  **Events (N)** | **Events (N)**  **at 12**  **months** | **Survival (%)**  **at 12**  **months** | **Events (N)**  **at 18**  **months** | **Survival (%)**  **at 18**  **months** | **HR (95%CI)** | **p-value** |
| --- | --- | --- | --- | --- | --- | --- | --- |
| **Gender** |  |  |  |  |  |  | 0.4903 |
| Male | 406(472) | 267(176) | 41.11% | 331(96) | 25.15% | 1 |  |
| Female | 389(448) | 228(186) | 46.87% | 304(100) | 26.99% | 0.92  (0.8 : 1.06) |  |
| **Age at first-line therapy** | 794(919) |  |  |  |  | 1.01  (1 : 1.02) | 0.0179 |
| **Primary tumor site** |  |  |  |  |  |  | 0.0003 |
| Intrahepatic | 395(460) | 233(196) | 47.59% | 301(111) | 29.93% | 1 |  |
| Extrahepatic | 175(204) | 108(81) | 43.94% | 135(49) | 28.74% | 1.02  (0.86 : 1.23) |  |
| Gallbladder | 177(196) | 124(60) | 34.63% | 161(20) | 12.66% | 1.48  (1.24 : 1.77) |  |
| Ampullary tumor | 46(56) | 28(24) | 47.55% | 36(16) | 32.03% | 0.81  (0.6 : 1.1) |  |
| **Disease status** |  |  |  |  |  |  | 0.0107 |
| Locally advanced | 171(206) | 92(99) | 53.36% | 126(59) | 33.92% | 1 |  |
| Metastatic | 619(708) | 400(260) | 41.10% | 505(136) | 23.73% | 1.29  (1.09 : 1.53) |  |
| **Period of first-line therapy** |  |  |  |  |  |  | 0.4903 |
| 2000-2009 | 269(274) | 168(104) | 38.30% | 206(65) | 24.13% | 1 |  |
| 2010-2013 | 339(374) | 203(157) | 44.61% | 269(87) | 25.58% | 0.91  (0.77 : 1.07) |  |
| 2014-2017 | 187(272) | 124(101) | 49.52% | 160(44) | 28.87% | 0.87  (0.72 : 1.06) |  |

**Table S3.** Contingency tables and summary output of univariate analysis for PFS, corrected for disease status and time in first-line therapy (n=410). p-value: Likelihood Ratio test p-value.

| **Characteristic** | **Overall**  **Events (N)** | **Events (N)**  **at 12**  **months** | **Survival (%)**  **at 12**  **months** | **Events (N)**  **at 18**  **months** | **Survival (%)**  **at 18**  **months** | **HR (95% CI)** | **p-value** |
| --- | --- | --- | --- | --- | --- | --- | --- |
| **Gender** |  |  |  |  |  |  | 0.9999 |
| Male | 180(187) | 172(9) | 5.31% | 177(4) | 2.52% | 1 |  |
| Female | 187(199) | 176(13) | 7.60% | 183(6) | 3.66% | 0.98  (0.8 : 1.21) |  |
| **Age at second line therapy** |  |  |  |  |  |  | 0.9999 |
| ≤60 years | 122(128) | 117(5) | 4.58% |  |  | 1 |  |
| 61 to 70 years | 129(135) | 122(8) | 6.84% | 128(2) | 2.02% | 0.99  (0.77 : 1.28) |  |
| ≥71 years | 116(123) | 109(9) | 8.46% | 110(8) | 7.57% | 0.93  (0.71 : 1.2) |  |
| **Primary tumor site** |  |  |  |  |  |  | 0.3611 |
| Intrahepatic | 192(203) | 185(8) | 4.50% | 191(2) | 1.33% | 1 |  |
| Extrahepatic | 73(78) | 65(9) | 13.82% | 67(7) | 10.91% | 0.87  (0.65 : 1.16) |  |
| Gallbladder | 75(77) | 73(3) | 4.59% |  |  | 1.29  (0.99 : 1.69) |  |
| Ampullary tumor | 26(27) | 24(2) | 9.78% |  |  | 0.74  (0.49 : 1.12) |  |
| **Biliary drainage** |  |  |  |  |  |  | 0.4443 |
| No | 293(308) | 275(21) | 7.47% | 286(10) | 3.65% | 1 |  |
| Yes | 74(78) | 73(1) | 2.31% |  |  | 1.3  (1 : 1.68) |  |
| **Surgery on primary**  **tumor** |  |  |  |  |  |  | 0.9999 |
| No | 181(195) | 172(11) | 6.65% | 177(6) | 3.76% | 1 |  |
| Yes | 186(191) | 176(11) | 6.27% | 183(4) | 2.46% | 0.95  (0.77 : 1.17) |  |
| **ECOG performance status** |  |  |  |  |  |  | 0.9999 |
| 0 | 159(167) | 148(12) | 7.89% | 155(5) | 3.48% | 1 |  |
| ≥1 | 184(194) | 178(7) | 4.46% | 181(4) | 2.68% | 1.16  (0.94 : 1.45) |  |
| **Clinical sensitivity** |  |  |  |  |  |  | 0.5474 |
| No | 118(123) | 111(9) | 8.18% | 115(5) | 4.74% | 1 |  |
| Resistant (PD within 90 days) | 52(55) | 50(2) | 4.88% |  |  | 1.1  (0.78 : 1.56) |  |
| Sensitive (PD after 90 days) | 197(208) | 187(11) | 6.12% | 193(5) | 2.93% | 1.3  (1.03 : 1.64) |  |
| **Period of second-line therapy** |  |  |  |  |  |  | 0.9999 |
| 2000-2009 | 105(107) | 99(6) | 6.43% | 99(6) | 6.43% | 1 |  |
| 2010-2013 | 134(138) | 124(10) | 7.92% | 133(1) | 1.15% | 0.89  (0.68 : 1.16) |  |
| 2014-2017 | 128(141) | 125(6) | 5.30% |  |  | 0.95  (0.73 : 1.24) |  |
| **Second-line therapy** |  |  |  |  |  |  | 0.9999 |
| COMBINED | 141(145) | 132(9) | 7% | 137(4) | 3.32% | 1 |  |
| FOLFOX or FOLFIRI | 85(87) | 82(3) | 4.12% |  |  | 1.07  (0.82 : 1.41) |  |
| MONO | 141(154) | 134(10) | 7.77% | 138(6) | 4.81% | 1.06  (0.84 : 1.34) |  |

**Abbreviations:** COMBINED, includes the following combination regimens: Cisplatin + gemcitabine, GEMOX, Gemcitabine + capecitabine and Others; FOLFOX, 5-fluorouracil/leucovorin + oxaliplatin; FOLFIRI, 5-fluorouracil/leucovorin + irinotecan; MONO, includes the following agents administered as monotherapy: Gemcitabine or Fluoropyrimidine.

**Table S4:** Contingency tables and summary output of the univariate OS analysis, corrected for the disease status and time in second-line therapy (n=410; see the text for further details).

Characteristic: variable taken into account; HR (95% CI): hazard Ratios with 95% Confidence Interval; p-value: Likelihood Ratio test p-value.

| **Characteristic** | **Overall**  **Events (N)** | **Events (N)**  **at 12**  **months** | **Survival (%)**  **at 12**  **months** | **Events (N)**  **at 18**  **months** | **Survival (%)**  **at 18**  **months** | **HR (95% CI)** | **p-value** |
| --- | --- | --- | --- | --- | --- | --- | --- |
| **Gender** |  |  |  |  |  |  | 0.9999 |
| Male | 172(198) | 139(41) | 25.91% | 153(24) | 16.86% | 1 |  |
| Female | 184(210) | 130(62) | 33.87% | 157(34) | 19.13% | 1  (0.81 : 1.23) |  |
| **Age at second line therapy** |  |  |  |  |  |  | 0.9999 |
| ≤60 years | 116(134) | 87(37) | 31.94% | 99(22) | 21.36% | 1 |  |
| 61 to 70 years | 127(145) | 95(34) | 29.20% | 113(16) | 13.97% | 1.19  (0.92 : 1.54) |  |
| ≥71 years | 113(129) | 87(32) | 29.37% | 98(20) | 19.35% | 1.07  (0.83 : 1.4) |  |
| **Primary tumor site** |  |  |  |  |  |  | 0.1594 |
| Intrahepatic | 184(218) | 134(59) | 34.01% | 159(33) | 19.57% | 1 |  |
| Extrahepatic | 73(83) | 55(23) | 31.55% | 60(15) | 24.35% | 1.16  (0.87 : 1.54) |  |
| Gallbladder | 72(79) | 62(11) | 16% | 67(6) | 9.05% | 1.52  (1.16 : 2.01) |  |
| Ampullary tumor | 26(27) | 17(10) | 38.19% | 23(4) | 16.40% | 0.95  (0.63 : 1.44) |  |
| **Biliary drainage** |  |  |  |  |  |  | 0.0848 |
| No | 284(328) | 208(90) | 32.62% | 243(52) | 19.85% | 1 |  |
| Yes | 72(80) | 61(13) | 19.67% | 67(6) | 10.47% | 1.44  (1.1 : 1.87) |  |
| **Surgery on primary**  **tumor** |  |  |  |  |  |  | 0.1594 |
| No | 184(210) | 149(43) | 24.67% | 168(23) | 13.68% | 1 |  |
| Yes | 172(198) | 120(60) | 35.66% | 142(35) | 22.57% | 0.79  (0.64 : 0.98) |  |
| **ECOG performance status** |  |  |  |  |  |  | 0.1014 |
| 0 | 154(179) | 109(52) | 35.26% | 131(29) | 20.44% | 1 |  |
| ≥1 | 182(203) | 146(40) | 23.23% | 164(20) | 12.71% | 1.32  (1.06 : 1.65) |  |
| **Clinical sensitivity** |  |  |  |  |  |  | 0.1594 |
| No | 117(132) | 86(38) | 32.65% | 98(24) | 22.37% | 1 |  |
| Resistant (PD within 90 days) | 50(55) | 40(11) | 25.38% | 45(6) | 14.36% | 1.32  (0.94 : 1.88) |  |
| Sensitive (PD after 90 days) | 189(221) | 143(54) | 29.81% | 167(28) | 16.36% | 1.38  (1.09 : 1.75) |  |
| **Second-line therapy**  **starting period** |  |  |  |  |  |  | 0.9999 |
| 2000-2009 | 111(112) | 85(26) | 24.26% | 94(17) | 16.02% | 1 |  |
| 2010-2013 | 135(146) | 96(42) | 32.25% | 113(24) | 19.24% | 0.87  (0.67 : 1.12) |  |
| 2014-2017 | 110(150) | 88(35) | 33.05% | 103(17) | 18.50% | 0.85  (0.65 : 1.12) |  |
| **Second-line therapy** |  |  |  |  |  |  | 0.2040 |
| COMBINED | 137(155) | 92(50) | 37.36% | 112(27) | 22.17% | 1 |  |
| FOLFOX or FOLFIRI | 71(89) | 59(17) | 26.67% | 65(10) | 17.29% | 1.12  (0.84 : 1.51) |  |
| MONO | 148(164) | 118(36) | 25.02% | 133(21) | 14.74% | 1.34  (1.06 : 1.7) |  |

**Abbreviations:** COMBINED, includes the following combination regimens: Cisplatin + gemcitabine, GEMOX, Gemcitabine + capecitabine and Others; FOLFOX, 5-fluorouracil/leucovorin + oxaliplatin; FOLFIRI, 5-fluorouracil/leucovorin + irinotecan; MONO, includes the following agents administered as monotherapy: Gemcitabine or Fluoropyrimidine.
